# Cognitive bias modification for social anxiety: protocol for a living systematic review of human studies and meta-analysis

**DOI:** 10.12688/wellcomeopenres.23278.1

**Published:** 2024-11-07

**Authors:** Jaycee Kennett, Claire Friedrich, Virginia Chiocchia, Simon E. Blackwell, Toshi Furukawa, Per Carlbring, Thomy Tonia, Ava Homiar, Simonne Wright, Kelvin Opiepie, Richardson Mojica, Paulina Schenk, Susan Michie, Janna Hastings, Hossein Dehdarirad, Claire Stansfield, James Thomas, Jennifer Potts, Georgia Salanti, Andrea Cipriani

**Affiliations:** 1 Department of Psychiatry, University of Oxford, Oxford, England, UK; 2 NIHR Oxford Health Biomedical Research Centre, Oxford Precision Psychiatry Lab, Oxford, UK; 3 Institute of Social and Preventative Medicine, University of Bern, Bern, Switzerland; 4 Department of Clinical Psychology and Experimental Psychopathology, Institute of Psychology, University of Göttingen, Göttingen, Germany; 5 Department of Health Promotion and Human Behaviour / School of Public Health, Kyoto University, Kyoto, Japan; 6 Department of Psychology, Stockholm University, Stockholm, Sweden; 7 Department of Psychiatry, Stellenbosch University, Stellenbosch, South Africa; 8 Global Lived Experience Advisory Board, GALENOS, Ogun State, Nigeria; 9 Global Lived Experience Advisory Board, GALENOS, Bacoor, Philippines; 10 Centre for Behaviour Change, University College London, London, UK; 11 Institute for Implementation Science in Health Care, Faculty of Medicine, University of Zurich, Zurich, Switzerland; 12 School of Medicine, University of St Gallen, St Gallen, Switzerland; 13 EPPI Centre, Social Research Institute, University College London, London, UK

**Keywords:** GALENOS, social anxiety, cognitive bias modification

## Abstract

**Background:**

Social anxiety is a heightened fear and discomfort in social situations which can be experienced in varying degrees of severity. Cases of elevated distress and impaired functioning and quality of life can lead to a clinical diagnosis of social anxiety disorder. Altering cognitive biases associated with social anxiety has been suggested as potentially beneficial; however, little is known about the comparative effectiveness of such interventions. The aim of this living systematic review is to examine the efficacy of cognitive bias modification for reducing social anxiety, including in people who have not been diagnosed with the disorder.

**Methods:**

We will search multiple electronic databases for randomised controlled trials evaluating the efficacy of cognitive bias modification for people diagnosed with social anxiety and people exposed to a simulated social stressor. The primary outcome will be change in social anxiety related symptoms; secondary outcomes will be changes in social functioning and quality of life and adverse events. Study selection, data extraction and risk of bias assessment will be done by at least two reviewers using pre-defined tools. We will synthesise data from people with social anxiety diagnosis and those subjected to a simulated social stressor separately using random effects meta-analyses. Heterogeneity will be evaluated by investigating characteristics of included studies. We will appraise the strength of the evidence for each outcome by reviewing the overall association, internal and external validity, and reporting biases. Where data allows, we will triangulate the evidence from both sources with a multidisciplinary group of experts. The review will begin in living mode and the database search will be rerun every three months to identify and integrate potential new evidence. We will co-produce this review with members of a global lived experience advisory board. This protocol was registered with PROSPERO (CRD42024601380) on 15.10.2024.

## Background and research questions

### Background

Anxiety is characterised by symptoms of worry and apprehension, psychological and autonomic arousal ( American Psychiatric Association, 2022). Social anxiety refers to these symptoms that happen specifically in the context of social situations ( Szuhany & Simon, 2022). This may involve everyday encounters such as having a conversation, eating or drinking with others, or performing in front of others ( *e.g.* giving a speech), and the feelings of anxiousness occur disproportionately to the actual threat posed by the social situation and the surrounding sociocultural context ( Morrison & Heimberg, 2013). These symptoms stem from a concern that the person will be judged negatively by others and may include feelings of humiliation, embarrassment. ( American Psychiatric Association, 2022; World Health Organisation, 2021). If social anxiety is persistent and causes significant impairment it may be diagnosed as social anxiety disorder (SAD). Individuals with SAD have been shown to preferentially attend to negative information about social situations, interpret ambiguous information as threatening, and display a bias towards remembering experiences more negatively. ( Stopa, 2009). These cognitive biases are thought to play a causal role in the development of SAD and its persistence ( Heimberg *et al.*, 2010).

Cognitive Bias Modification (CBM) interventions aim to alter these biases via simple repetitive training procedures and by doing so improve symptoms of mental disorders ( Vrijsen *et al.*, 2024). CBM is an umbrella term which includes interventions which specifically aim to alter attentional biases (CBM-A), or interpretation biases (CBM-I). CBM intervention studies for SAD have shown mixed results; while some randomised controlled trials (RCTs) have found CBM-A to be effective in reducing SAD symptoms ( Amir *et al.*, 2009; Schmidt *et al.*, 2009), other studies have failed to replicate these findings ( Julian *et al.*, 2012). Early claims of promise for attention bias modification were soon followed by criticisms of the evidence base ( *e.g.*, Kruijt & Carlbring, 2018). There is evidence that certain kinds of CBM interventions may be effective in reducing symptoms for a range of disorders such as depression and anxiety ( Fodor *et al.*, 2020), and alcohol addiction ( Boffo *et al.*, 2019).

It remains unclear as to which subtypes of CBM are more effective than others for specific disorders. Cognitive behavioural therapy (CBT) remains the most often recommended first line treatment for SAD ( Pelissolo *et al.*, 2019); CBT consists of 1:1 sessions with a mental health professional, usually once a week for a minimum of six weeks, contrasting with CBM which can be delivered without the guidance of a mental health professional and specifically targets a cognitive bias. However, there are numerous logistical and financial barriers to accessing psychological services such as CBT, particularly in the Global South. As CBM can be delivered digitally and without instruction, it represents a potentially cost-effective intervention that could be used in countries in which access to professional psychological support is limited. As people with SAD are less likely to access in-person mental healthcare due to the nature of their disorder, CBM potentially offers an effective method of providing support to them ( Goetter *et al.*, 2020). Moreover, CBT is now a complex intervention involving different techniques, each having their foundation in behaviourist or cognitive theories, which make their further improvement also complex. By contrast, CBM is more mechanistic and founded in experimental psychology and therefore may lend itself to more straightforward refinement ( Holmes *et al.*, 2018).

A better understanding of the CBM literature on SAD is crucial to improving these interventions, as is disentangling both the effectiveness of different subtypes of CBM and its mechanisms. Understanding more about which subtypes are most effective and by examining the mechanisms by which cognitive biases can be changed most effectively will improve the targeting of interventions and provide a better starting point for future CBM research.

The effects of CBM have also been investigated via altering cognitive biases in healthy volunteers and measuring emotional or physiological responses to a social stressor ( *e.g.* Vassilopoulos *et al.*, 2015). This enables investigation of the link between the modulation of cognitive biases and emotional and physical responses in a situation where social anxiety is induced ( MacLeod & Mathews, 2012).

This living systematic review (LSR) will use MacLeod and Mathews’s (2012) definition of cognitive bias, defining it as “a systematic selectivity in information processing that operates to favour one type of information over another" (p2). CBM will be used as an umbrella term for interventions that target any cognitive bias, defining it as directly altering a specific cognitive bias by repeatedly exposing individuals to tasks that promote predetermined patterns of selective processing ( MacLeod & Mathews, 2012). To examine the association between CBM and symptoms of social anxiety, it will draw on two sources of evidence; a) individuals with diagnosed SAD (hereafter referred to as the clinical source of evidence) and b) people without any mental health condition. This will include people who may have elevated levels of social anxiety/sub clinical social anxiety but have not been diagnosed with SAD, and individuals not recruited for studies based on having SAD. These two sources of evidence will each be examined using separate network meta-analyses and will be combined through triangulation.

### Review objectives

- To review the evidence of the effects of CBM on social anxiety symptoms in people with social anxiety disorder.
- To review the evidence of the effects of CBM on social anxiety in people without any mental health condition.
- To describe the potential mediators of the effects of CBM on the symptoms of social anxiety.

### Research questions

1. What are the effects of CBM on reducing symptom severity in individuals with SAD?
2. What are the effects of CBM on changing social anxiety in individuals not diagnosed with any mental health condition?
3. What mediates the effects of CBM on social anxiety?

## Methods

### Study inclusion and exclusion criteria

The population, intervention, comparator, and outcomes that we will include and exclude for both sources of evidence are detailed in Table 1 and Table 2.

**Table 1.** Study inclusion and exclusion criteria for source of evidence: individuals with SAD.

*Study design*	*We will include:* • Randomised controlled trials • Studies published in any language and in any year
*Population*	*We will include:* • Participants with a clinical diagnosis of social anxiety disorder or above-threshold symptoms on any standardised scale used to diagnose social anxiety disorder • Participants of any age *We will exclude:* • Participants with social anxiety disorder as a secondary diagnosis and who were recruited based on another mental health diagnosis • Participants who are undergoing any other psychological treatment during the study period
*Experimental* * interventions/exposures*	*We will include:* • An intervention of CBM, structured and designed to alter a cognitive bias related to social situations. We will accept any number of sessions or any delivery format • Interventions which include multi-component CBM *We will exclude:* • CBM as augmentation of a pharmacological or psychological intervention which occur concurrently
*Control interventions/* *non-exposures*	*We will include:* • Inactive controls such as a) waitlist control or b) no intervention • Non-specific controls such as a) sham CBM, b) placebo training, c) attention control condition, d) neutral control condition (i.e. a task that does not involve any CBM, or e) other interventions that aim to control for aspects of the CBM intervention such as time spent completing tasks or interaction with the experimental setup, but which are not intended to affect cognitive biases • Negative controls (where participants are trained towards the opposite cognitive bias to the intervention) *We will exclude:* • Any form of current psychotherapy (defined as a directed psychological intervention designed to improve distress)
*Outcomes*	Studies will be included irrespective of outcomes reported. Primary outcome: • Change in social anxiety severity from pre to post intervention Secondary outcomes: • Acceptability (number of drop-outs due to any reason) • Tolerability (number of drop-outs due to adverse events) • Number of participants reporting any adverse events • Number of participants reporting each specific adverse event. To mitigate variability in how adverse events are reported we will use the Medical Dictionary for Regulatory Activities to harmonise and organise terminology related to adverse events • Change in cognitive bias measured as self-report measures or attentional or interpretive bias tasks • Clinical global impression of improvement • Change in diagnostic status (no longer meeting diagnostic criteria) • Change in general anxiety levels ( *e.g.* GAD-7, BAI) • Change in general depression levels ( *e.g.* PHQ-9, BDI-II) • Change in quality of life/measures of subjective wellbeing • Change in symptoms of social functioning

**Table 2.** Study inclusion and exclusion criteria for the source of evidence: individuals without any mental health condition.

*Study design*	*We will include:* • Randomised controlled trials • Studies published in any language and in any year
*Population*	*We will include:* • Participants who have not been recruited based on having a diagnosis of SAD • Participants of any age • Participants exposed to an analogue social stressor during the study (i.e. a task or situation that aims to induce mild social anxiety) *We will exclude:* • Participants recruited for having a specific physical or mental health diagnosis • Participants who are undergoing any other psychological treatment during the study period
*Experimental* * interventions/exposures*	*We will include:* • An intervention of CBM, structured and designed to alter a cognitive bias relating to stressful social situations. We will accept any number of sessions or any delivery format. • Interventions which include multi-component CBM *We will exclude:* • CBM as augmentation of a pharmacological or psychological intervention which occurs concurrently
*Control interventions/* *non-exposures*	*We will include:* • Inactive controls such as waitlist control or no intervention • Non-specific controls such as a) sham CBM, b) placebo training, c) attention control condition d) neutral control condition (i.e. a task that does not involve any CBM or e) other interventions that aim to control for aspects of the CBM intervention such as time spent completing tasks or interaction with the experimental setup, but which are not intended to affect cognitive biases • Negative controls (where participants are trained towards the opposite cognitive bias to the intervention) *We will exclude:* • Any form of current psychotherapy (defined as a directed psychological intervention designed to improve distress)
*Outcomes*	Studies will be included irrespective of outcomes reported. Primary outcomes: • Change in social anxiety-related symptoms, defined below Secondary outcomes: • Acceptability (number of drop-outs due to any reason) • Tolerability (number of drop-outs due to adverse events) • Number of adverse events • Number of participants with a specific adverse event to mitigate variability in how adverse events are reported we will use the Medical Dictionary for Regulatory Activities to harmonise and organise terminology related to adverse events • Change in cognitive bias

### Study identification

The search strategy will be defined in collaboration with the search team and informed by prior research ( Fodor *et al.*, 2020; Martinelli *et al.*, 2022; Spijker *et al.*, 2023). The ontology team will be informed of the search strategy and will help identify additional search terms where possible and relevant. The resulting search strategy will also inform the scope of the ontology ( Schenk *et al.*, 2024).

The primary approach in developing the search strategy will focus on searching titles, abstracts, keywords and database-controlled vocabulary on the concepts of Cognitive Bias Modification (CBM) and studies with a randomised experimental design. To enhance sensitivity, a range of terms for CBM will be used. In addition, to supplement these terms, terms are used to find research using cognitive bias detection methods within the context of social anxiety.

In this initial iteration, we will search the following databases covering health, psychology and multi-disciplinary research fields: CENTRAL (Cochrane Library), Embase (OVID), MEDLINE (OVID), PsycINFO (OVID), Scopus, Web of Science (SCI, SSCI, ESCI and the related conference and book indexes). LILACS, WHO Index Medicus, the trial registries (ClinicalTrials.gov, ScanMedicine, WHO-ICTRP) will also be searched. Searches will be adapted and simplified where necessary. Dissertation abstracts will be excluded. In addition, reference lists of key relevant reviews known the to review team will be manually checked. See the extended data for the search strategy for Medline (OVID) as an example ( Kennett *et al.*, 2024). This protocol was registered on PROSPERO (CRD42024601380) on 15.10.24.

### Study selection

Once the searches have been completed, records will be imported into EPPI-Reviewer ( Thomas *et al.*, 2020) and duplicates will be removed. The titles and abstracts of the identified records will be examined by two members of the review team who will independently mark each one as eligible or ineligible. Disagreements between the two will be resolved through discussion or in consultation with a third reviewer. The full texts of all records deemed eligible will then be retrieved and screened by two members of the review team, disagreements will be resolved in the same way. In the case that a full text of an eligible abstract cannot be located, corresponding authors will be contacted. In the event of no response, a follow up email will be sent one month later. If no response is received, the study will be included, and we will continue to attempt to contact the authors in further iterations of the review. The selection process will be recorded and reported in a PRISMA flowchart ( Page *et al.*, 2021a). Excluded studies will be reported along with reasons for exclusion in a 'Characteristics of excluded studies' table.

### Outcomes and prioritisation

***Source of evidence: participants with SAD***

**Primary outcomes**

Our primary outcome of interest will be the change in social anxiety symptom severity from pre- to post-intervention, measured with validated clinician-rated or self-reported measures ( *e.g.* Liebowitz Social Anxiety Scale (LSAS, Liebowitz, 1987).

**Secondary outcomes**

- We will seek data for the **acceptability** of CBM (measured by the number of total dropouts and the number of dropouts due to adverse events),
- We will seek data on the **tolerability** of CBM (dropouts due to adverse events), as well as details of the reasons given for dropouts due to any reason and details of any adverse events reported to occur in CBM studies.
- We will also seek to examine data on **change in cognitive bias** following the intervention, either self-reported or measured on attentional or interpretive tasks.
- Additionally, we will include **quality of life** as a secondary outcome as individuals with social anxiety often report poor quality of life ( Dryman *et al.*, 2016). This was prioritised by our Global Lived Experience Advisory Board (GLEAB), as an important element of social anxiety to consider.
- We will also seek data on **clinical global impression of improvement**, **change in participants diagnostic status** (whether, following treatment, they were judged not to have SAD) and **levels of depression** and **levels of general anxiety**.

***Source of evidence: participants without any mental health condition***

**Primary outcomes**

The primary outcome for the non-clinical source of evidence will be change in social anxiety related symptoms via observer or self-reported scales. The most recent versions of the Diagnostic and Statistical Manual of Mental Disorders (DSM-5-TR) and the International Classification of Diseases (ICD-11) characterises social anxiety disorder as feelings of fear, beliefs that one’s actions will be negatively evaluated by others, and avoidance of social interactions ( American Psychiatric Association, 2022; World Health Organisation, 2021). In their systematic review of social anxiety measures, Wong *et al.* (2016) echo the importance of these domains, in addition to reporting that social anxiety measures also seek to capture physiological elements, feelings of a racing heart, increased perspiration, and faster breathing. As research on populations without any mental health conditions is unlikely to have utilised clinical social anxiety scales, to capture symptoms related to social anxiety, this systematic review will seek data from measures that are intended to capture the following areas:

- Avoidance behaviours
- Feelings of fear
- Belief in future negative evaluation
- Heart rate variability
- Blood pressure
- Skin conductance

Where it is unclear whether a particular measure relates to social anxiety-related symptoms, content experts will be consulted. However, where a nonclinical study does report and use measures pertaining to social anxiety symptom severity, we will prioritise these.

**Secondary outcomes**

For the evidence on individuals not diagnosed with any mental health condition, the secondary outcomes will be the same as for the SAD source of evidence, detailed above although we will not seek data on global clinical impression improvement, quality of life and subjective wellbeing, general anxiety or depression levels, or changes in social functioning.

**Prioritisation of effect measures**

It is anticipated that measures used and reported across trials will vary, where a trial reports more than one measure of the same outcome we will follow a predefined hierarchy for selecting the measure, as follows:

- Where a trial reports a measure as their primary outcome we will prioritise this;
- If they do not clarify a primary outcome, we will select the one which is not self-reported;
- If all measures are self-reported, we will select the one with best psychometric properties;
- If they have similar psychometric properties, we will select the one which has most commonly been used in other studies including in our review.

**Mediators**

To address research question three; examining potential mediators influencing effects of CBM on social anxiety, we will extract data on mediators in the included studies as a secondary outcome and describe these narratively.

### Data extraction

Data will be extracted using EPPI-Reviewer ( Thomas *et al.*, 2020). Before extraction begins, the ontology team will review the data extraction form to identify relevant ontology categorisations to support data extraction. The form will be piloted and if subsequent revisions are required, these will be made prior to beginning data extraction. Two reviewers will independently extract data using the final extraction form and any differences will be reconciled through discussion or involvement of a third reviewer. We will extract:

- Study duration (in months)
- Year of study completion
- Date of publication
- Conflicts of interest
- Population characteristics: number of participants randomised (total, per arm), age, gender as reported in study, ethnicity, medical diagnoses and treatment information
- Intervention characteristics: number of sessions, setting where the intervention takes place, how the intervention is delivered, duration of intervention, and type of cognitive bias targeted
- Comparator: whether the comparator is a placebo or inactive control, if the comparator is a non-specific or negative control, we will extract the same level of detail as for the intervention
- Outcomes

***Extraction of outcome data***

For continuous outcomes, data will be extracted as means and standard deviations at baseline and post-intervention timepoints, if available, or as change from baseline. If standard deviation is not reported, it will be calculated manually from standard error. If standard error is also missing, it will be calculated from reported test statistics, confidence intervals (CIs) or alternative reported distribution ( *e.g.* median/range). If a study uses more than one outcome measure (continuous and dichotomous), we will prioritise the continuous outcome.

Where studies report number of events ( *e.g.* dropouts or number of participants reporting adverse events) we will extract the numbers reported. Where studies report these as percentages, we will convert these to absolute numbers. Any participant who is randomised but does not complete the final assessment in the study will be considered a dropout due to any reason.

When there is missing outcome data, we will examine trial registries and clinical study reports. If this is not possible, we will contact the authors and if there is no reply, we will send one follow up email one month later. Where studies have used methods that account for missing data, we will give preference to mixed models of repeated measurement (MMRM) and multiple imputations, followed by last observation carried forward (LOCF) and observed cases. If a study reports both completer and imputed analyses, we will prioritise imputed data. In the case of a study reporting multiple time points, we will prioritise the data reported at the end of the intervention. If this is not available, we will extract the next closest post-intervention timepoint.

**Data extraction for mediators**

Where mediators are reported in the included studies ( *e.g.* levels of attention) we will extract the mediator, the relevant outcome, the measure used to examine the mediating effect and report the association found using the exact statements in the study regardless of statistical significance. Data will be extracted on any mediator. Previously reported mediators of CBM are:

- Change in attentional and interpretive biases ( Nieto & Vasquez, 2021; Price *et al.*, 2016)
- Levels of trait anxiety ( Salemink *et al.*, 2010)
- Change in mood ( Salemink *et al.*, 2010)
- Contingency learning ( Beadel *et al.*, 2014)

***Risk of bias***

The Risk of Bias 2 (RoB 2) tool will be used to assess risk of bias for randomised controlled trials for each outcome ( Sterne *et al.*, 2019). The overall risk of bias will be graded as: low risk, if no domains are rated high risk and no more than one domain is rated some concerns; high risk, if any domain is rated high risk; and some concerns, if more than one domain is rated as having some concerns and no domains are rated high risk. The risk of bias for all outcomes of all studies will be assessed by two reviewers and any disagreements settled by discussion or by a third reviewer. The risk of bias assessments for each outcome will inform the certainty of the evidence, and presented in the summary of evidence table, as well as in traffic light plots.

### Data analysis and synthesis

If different scales are used to measure the same outcome, we will standardise the direction for the outcome using score inversion. For example, this may occur when the Situational Confidence Questionnaire ( Annis & Graham, 1988) is used to measure social anxiety, where a higher score indicates improvement in social anxiety symptoms. If data from this scale were to be extracted in this review, the direction of scores would be standardised to have the same direction as the a priori selected outcome: social anxiety symptom severity, where higher scores indicate greater symptom severity.

To report the relative effects of CBM for continuous outcomes, we will calculate mean difference if the included studies have all used the same scale. If the included studies use different scales, we will calculate standardised mean difference (SMD). For dichotomous outcomes such as adverse events, odds ratio (OR) will be used as a measure of effect. 95% confidence intervals will be reported for all outcomes.

***Pairwise meta-analysis***

We will perform a quantitative synthesis via a pairwise meta-analysis using a frequentist random effects model for all CBM interventions versus control conditions. Heterogeneity will be assessed by visual inspection of forest plots and presented by the 95% prediction intervals. REML will be used to estimate the variance in heterogeneity τ, and 95% confidence intervals will be corrected using a Hartung Knapp correction should more than five studies contribute to the analysis.

***Network meta-analysis***

We expect there to be significant heterogeneity within the control conditions used in CBM trials, particularly among sham conditions ( Fodor *et al.*, 2020). However, previous reviews have been critiqued for making the assumption that all sham conditions, despite involving different components, could be adequately lumped as one control group ( Blackwell, 2020). CBM trials often utilise sham conditions that attempt to match the intervention group as closely as possible, however qualitative differences between these conditions may pose a problem when combining them for pairwise meta-analysis ( Blackwell *et al.*, 2017). If enough data for the same outcome is available, we will conduct a quantitative synthesis using a random effects network meta-analysis (NMA) model, separating the different control conditions and the different CBM approaches. We will check that clinical and methodological characteristics that could act as effect modifiers (see investigation of heterogeneity below) are distributed similarly across comparisons to ensure that the assumption of transitivity is not violated.

We will assume a common heterogeneity parameter (τ ^2^) across treatment comparisons in the NMA random effects model. Relative treatment effects will be reported in forest plots and league tables, with treatments ordered according to ranking based on the surface under the cumulative ranking curve (SUCRA) ( Salanti *et al.*, 2011). Data analysis will be conducted in R statistical software using the packages meta ( Balduzzi *et al.*, 2019), netmeta ( Balduzzi *et al.*, 2023), and NMA ( Noma *et al.*, 2024).

We have specified a list of expected CBM interventions that will be included based on previous reviews (see Table 3).

**Table 3.** List of expected interventions.

Abbreviation	Intervention
IBM / CBM-I	Interpretive Bias Modification
ABM / CBM-A	Attention Bias Modification
MBM	Memory Bias modification
CBM-MC	Multi-Component CBM
CBM-App	Cognitive Bias Modification- Appraisal
AIM	Attention Interpretations Modification
VRET	Virtual Reality Exposure Therapy

However, as there are often novel adaptations of CBM paradigms (see Martinelli *et al.*, 2022), we expect this list will not be exhaustive. If a novel variant of CBM is reported, the research team will adjudicate the extent to which CBM variants can be lumped into nodes or be a stand-alone node, considering the methodological and clinical implications of doing so. An example of a novel variant of CBM is in the study by Rohrbacher *et al.* (2014) where standard CBM-I was compared to a variant of CBM-I which included participants taking part in an image generation task as part of the intervention. This adjudication will be done after data have been collected, but before data have been analysed and no researchers involved in data extraction will be involved in the adjudication. When important uncertainty or disagreement persist among the research team, we will decide on the primary model and use the remaining as sensitivity analyses, before we conduct the meta-analyses.

Similarly, we have specified a list of control interventions that we expect to find and that will form different nodes in our network (see Table 4).

**Table 4.** List of expected control conditions.

Control condition
Opposite ABM
Opposite CBMI
Sham
Waitlist
Treatment as usual

To ensure the network is both methodologically valid and clinically relevant, we will determine to what extent heterogenous control interventions should be lumped via the adjudication process described above, after collection of data but without knowledge of the outcome. If a NMA is conducted, we will assess inconsistency in the network using both local and global methods ( Salanti, 2012), respectively the SIDE (separating-indirect-evidence-from-direct-evidence) test and the design-by-treatment interaction test ( Dias *et al.*, 2010; Higgins *et al.*, 2012) 

### Investigation of heterogeneity

We will investigate potential sources of heterogeneity and inconsistency by exploring the following effect modifiers in subgroup analyses:

- At least one of the authors has allegiance bias (is involved in the CBM intervention that is tested)
- Delivery mode of CBM (in person/online/via mobile)
- Number of sessions (a posteriori categorisation)
- Cognitive bias targeted; interpretive, attentional, or memory bias

### Sensitivity analysis

We will perform a sensitivity analysis for the primary outcome excluding studies with high risk of bias. We will also use sensitivity analysis, limiting the clinical group to individuals diagnosed with SAD by a medical professional, not just those with above threshold symptom scores.

### Synthesis of mediators

The details of any mediators of CBM on social anxiety symptoms in the included studies will be descriptively reported.

### Reporting bias

Reporting bias for the pairwise meta-analysis will be assessed using the Risk of Bias due to Missing Evidence tool ( Page *et al.*, 2021b). Should an NMA be conducted, we will assess reporting bias using the Risk of Bias due to Missing Evidence in Network meta-analysis (ROB-MEN) tool ( Chiocchia *et al.*, 2021).

### Summary of the evidence

We will assess the certainty of the evidence for each outcome for each source of evidence (individuals with and without SAD ). Summary of Evidence (SoE) tables will be produced for each outcome for each source of evidence with outcomes as rows and domains of confidence as columns (see Table 5 and Table 6). The confidence in the evidence will be assessed by a single reviewer and checked by a second reviewer. Disagreements will be resolved by discussion or involvement of a third reviewer. Should a pairwise meta-analysis and an NMA be conducted, we will prioritise the pairwise meta-analysis.

**Table 5.** Example summary of Evidence (SoE) table for the source of evidence: individuals diagnosed with SAD.

Outcome	Source of evidence	Timepoint	Summary of the association	Bias due to study limitations	Bias due to reporting bias	Bias due to indirectness:	Bias due to other reasons
The effects of CBM on outcome X	Studies on individuals diagnosed with SAD	End of study	Number of studies, total number of participants. Numerical summary of meta-analysis with point estimate, 95% confidence and prediction intervals.	Number of studies rated low, some concerns, and high in RoB 2 tool, expected direction of bias ( *e.g.* over- or underestimation of the true effect). Assessment of the internal validity of the findings using the sensitivity analysis using only studies with low risk of bias.	Assessment of the impact of reporting bias on the size and direction of the bias based on findings using the ROB-ME tool ( Page *et al.*, 2021b)	Assessment of the potential impact of bias and its direction on the magnitude and size of the effect due to the included studies not capturing the intended population, intervention, comparator, and outcome.	Assessment of the impact of any other sources of bias. We will follow a thorough review process to minimise other sources of bias.

**Table 6.** Example summary of Evidence (SoE) table for the source of evidence: individuals not diagnosed with any mental health condition.

Outcome	Source of evidence	Timepoint	Summary of the association	Bias due to study limitations	Bias due to reporting bias	Bias due to indirectness:	Bias due to other reasons
The effects of CBM on outcome X	Studies on individuals without any mental health condition	end of study	Number of studies, total number of participants. Numerical summary of meta-analysis with point estimate, 95% confidence and prediction intervals.	Number of studies rated low, some concerns, and high in RoB 2 tool, expected direction of bias ( *e.g.* over- or underestimation of the true effect). Assessment of the internal validity of the findings using the sensitivity analysis using only studies with low risk of bias.	Assessment of the impact of reporting bias on the size and direction of the effect based on findings using the ROB-ME tool ( Page *et al.*, 2021b)	Assessment of the potential impact of bias and its direction on the magnitude and size of the effect due to the included studies not capturing the intended population, intervention, comparator, and outcome.	Assessment of the impact of any other sources of bias. We will follow a thorough review process to minimise other sources of bias.

## Triangulation of the evidence from living systematic reviews

It has been argued that SAD can be distinguished from feelings of nervousness and shyness in social situations neurobiologically ( Liu *et al.*, 2015), cognitively ( Lavoie *et al.*, 2014), and in terms of quality of life ( Stein & Kean, 2000). As such, we will consider the two sources of evidence in this living systematic review– people diagnosed with SAD and those without a mental health condition –sufficiently different to potentially warrant triangulation methods to consider their results together. Where there is sufficient evidence for one outcome from both sources and the sources’ respective errors and biases are deemed to be unrelated, we will discuss these outcomes in a triangulation meeting. This will include members of the review team, methodological experts, and experts in CBM and SAD. The SoE tables from both sources of evidence will be used in the triangulation meeting to draw an overall conclusion on the effect of CBM on social anxiety, based on the direction of the results and the strength of the evidence.

## Updating the systematic review and ending the living mode of the review

### Updating the living systematic review

We will conduct searches every three months to identify new evidence that is relevant to the review; the number of databases may be reduced for the update searches, if some databases do not provide a unique yield of relevant references in the first search. As we find new evidence, the authors of the last iteration of the review will assess the evidence and based on its potential to substantially change the overall findings, we will incorporate it into the review. We will report all updates using the versioning system of F1000 (Wellcome Open Research), highlight the new evidence added, and specify when each update has been performed. After the conclusion of the initial version of the systematic review, the methods will be reconsidered to judge their suitability and efficiency in practice. If deemed appropriate, the methodological approaches of data extraction and synthesis will be adapted accordingly, and any changes will be documented. We will also investigate the use of machine learning (including large language models) for automating some of the study selection (and possibly data extraction, should technology advance sufficiently) tasks once the review is in living mode. Where we can demonstrate that we can save manual effort without compromising the reliability of the review, we will use these new technologies to make our overall workflows more efficient. If deemed appropriate by the review team, inclusion and exclusion criteria will be adapted as necessary with changes being reported on our versioning system. Any deviations from this protocol will be reported with justification.

### Ending the living mode of the review

At each triangulation meeting, the decision to continue the living mode of the review will be discussed, and for which outcomes it should continue. If it is decided that it is unlikely that new evidence changing the results of the review will be published and that the research aims of the review have been fulfilled, the review will exit living mode. If there is not enough evidence to call for a triangulation meeting, the review will continue in living mode with its planned searches.

## Co-production aspects

This protocol has been written in collaboration with people with lived experience. The question was selected by members of GALENOS’ Global Lived Experience Advisory Board (GLEAB) during a prioritisation exercise, in which several questions were ranked against a criterion that was decided upon by the group. Two members of the GLEAB are part of the author team for this review and have contributed to the writing of this protocol, including: the formulation of the research aims, the outcomes of importance, and the design of the review.

The lead author was provided with guidance on how to effectively include people with lived experience in their research, while members of the GLEAB were provided with training on systematic reviews.

Considering the complexity and multidimensionality of the review topic, we will establish a schedule of regular team meetings and foster effective communication within the GALENOS project. The primary objective of these initiatives is to facilitate a shared understanding, promote the transferability of knowledge, encourage the exchange of ideas and perspectives, and identify the distinct needs of various stakeholders. By implementing these measures, we aim to create an environment where all stakeholders have equal standing and can actively contribute to the collaborative production of the review.

## Dissemination of information

We plan to publish the review on the GALENOS website and on Wellcome Open Research. A plain language summary will accompany the review. We will use social media outlets to publicise the results and will write blog posts that will be available on the GALENOS website. We will also include the results in the quarterly Research Roundup newsletter that MQ issues. We hope to present GALENOS at the World Congress of Biological Psychiatry as well as other conferences.

## Study status

The study status at the date of submission 15.20.2024 is reported below.

### Preliminary searches

Started, not completed

### Piloting the study selection process

Not started

### Piloting the study selection process

Not started

### Full searches

Not started

### Full screening of search results against eligibility criteria data extraction

Not started

### Risk of bias or quality assessment

Not started

### Data synthesis

Not started

## Ethics and consent

Ethical approval and consent were not required.

## Data Availability

No data are associated with this article. OSF: Cognitive bias modification for social anxiety: protocol for a living systematic review of human studies and meta-analysis. https://doi.org/10.17605/OSF.IO/2W69Z ( Kennett *et al.*, 2024). OSF: PRISMA-P checklist for ‘Cognitive bias modification for social anxiety: protocol for a living systematic review of human studies and meta-analysis’. https://doi.org/10.17605/OSF.IO/2W69Z ( Kennett *et al.*, 2024). Data are available under the terms of the Creative Commons Attribution 4.0 International license (CC-BY 4.0).

## Software availability

We will use EPPI-Reviewer for study selection and data extraction ( Thomas *et al.*, 2020, available at EPPI-Reviewer: systematic review software).
