# Supplementary material for: Cognitive bias modification for social anxiety: protocol for a living systematic review of human studies and meta-analysis
Source: Wellcome Open Res. 2025 May 23;9:657. Originally published 2024 Nov 7. [Version 2] doi: 10.12688/wellcomeopenres.23278.2 (PMC12149809; doi:10.12688/wellcomeopenres.23278.2)
Supplement: Peer review history

# Reviewer report: Reviewer response for version 2

**DOI:** 10.21956/wellcomeopenres.26799.r124418

**Published:** 2025-06-09

**Authors:** Yujia Peng

**Affiliations:** 1 Peking University, Beijing, China

The current study protocol presented an exciting direction focusing on cognitive bias modification for social anxiety (SA). The aim of this living systematic review is to examine the efficacy of cognitive bias modification for reducing SA.The current study protocol presented an exciting direction focusing on cognitive bias modification for social anxiety (SA). The aim of this living systematic review is to examine the efficacy of cognitive bias modification for reducing SA.

 The study design is overall clear and feasible. The theoretical contribution is relatively clear. As previous reviewers mentioned, a few questions remain to be answered:

  

1. Living systematic review, can the authors clearly define this term? And explain how does a living systematic review benefit the field? The feasibility and necessity should be more clearly addressed.
2. Is conducting reviews and updates every 3 months too frequent? Would an interval of 6-12 months be more appropriate?
3. It is recommended to provide more details about the CBM intervention measures, such as stimulus presentation methods, number of training sessions, duration, etc., to help readers better understand its advantages over CBT.
4. The method of categorizing CBM interventions into 7 subcategories requires explanation and definition. It would be preferable to allocate the corresponding studies to their respective subcategories.

Is the study design appropriate for the research question?

Yes

Is the rationale for, and objectives of, the study clearly described?

Yes

Are sufficient details of the methods provided to allow replication by others?

Yes

Are the datasets clearly presented in a useable and accessible format?

Not applicable

Reviewer Expertise:

computational psychiatry, depression and anxiety, neuroimaging, social cognition

I confirm that I have read this submission and believe that I have an appropriate level of expertise to confirm that it is of an acceptable scientific standard.

# Reviewer report: Reviewer response for version 2

**DOI:** 10.21956/wellcomeopenres.26799.r124066

**Published:** 2025-06-03

**Authors:** Kate Clauss

**Affiliations:** 1 VA Portland Healthcare System, Portland, Oregon, USA

The authors were responsive to feedback and the methods are clearer as a result. I do think it will be challenging to update the living review every 3 months, but that schedule could be modified as needed. Looking forward to seeing the authors bring data repositories to the CBM space.

Is the study design appropriate for the research question?

Yes

Is the rationale for, and objectives of, the study clearly described?

Partly

Are sufficient details of the methods provided to allow replication by others?

Yes

Are the datasets clearly presented in a useable and accessible format?

No

Reviewer Expertise:

My research focuses on cognitive dysfunction in PTSD and related conditions with an emphasis on attentional bias and cognitive bias modification. I also work on several large study-level data repositories (i.e., PTSD Repository, Suicide Prevention Trials Database), which are similar to the living review in the proposed study.

I confirm that I have read this submission and believe that I have an appropriate level of expertise to confirm that it is of an acceptable scientific standard.

# Reviewer report: Reviewer response for version 1

**DOI:** 10.21956/wellcomeopenres.25657.r110652

**Published:** 2024-12-12

**Authors:** Kate Clauss

**Affiliations:** 1 VA Portland Healthcare System, Portland, Oregon, USA

Thank you for the opportunity to review: “Cognitive bias modification for social anxiety: protocol for a living systematic review of human studies and meta-analysis.” I think this is an exciting addition to the field of CBM. Below are several suggestions to strengthen the description of the project and the utility of the living review.

 ABSTRACT

- I believe the second background and method section is a version of the abstract for a lay reader. Could this be labelled as such?
- In the introduction, there is considerable emphasis on the different subtypes of CBM and which would be most appropriate for social anxiety, but this is not mentioned in the abstract. Please make these consistent.

INTRODUCTION

- Could the authors define interpretation and attentional biases? There are examples in the first paragraph, but these are not clearly labelled. This would provide more context for the second line in paragraph two where they introduce CBM-A and CBM-I, which are designed to target attentional and interpretation biases.
- Given that most cognitive models also suggest that memory biases contribute to psychopathology, it maybe also be useful to reference this in the intro. Particularly, given that Memory Bias modification is one of the types of CBM the authors intend to use and memory bias is being investigated as a source of heterogeneity.
- Additional information on what CBM interventions are, would be beneficial. For example, describing the format (i.e., presenting stimuli via the computer), number of trials, duration of a training session, and typical number of sessions would provide the reader with more context. Particularly, given that the next paragraph speaks to the advantages of CBM over CBT.
- The first sentence in paragraph three was misleading. I thought the paragraph would be about whether CBM-A vs. CBM-I was more beneficial for social anxiety. Please reword.
- In paragraph 4, the authors note that an important next step is understanding which subtypes are most effective and which mechanisms underlie changes in cognitive biases; however, mechanisms were not mentioned in the abstract as one of the primary outcomes for the systematic review/meta-analysis – this would be helpful to include. Similarly, the differential effect of the subtypes of CBM is not included in the review objectives or research questions, which was surprising after the emphasis on this in the intro.
- I think the fifth paragraph would be more appropriate at the end of paragraph two where evidence for the efficacy of CBM is discussed.
- The benefits of a living systematic review could be more clearly delineated. This is an exciting next step for our field and having such a resource could greatly increase the speed with which future meta-analyses on CBM in social anxiety are conducted. Will the living review be available to other researchers after a period?
- The types of CBMs the authors intend to include in the meta-analysis (Table 3) are actually quite a bit more diverse than we are led to believe in the intro (i.e., only CBM-A and CBM-I are discussed). Will each be analyzed separately, or grouped into categories (e.g., CBM-A, CBM-I, CBM-memory, etc.)?

METHOD

- I noticed that the authors plan to include studies published in any language. Is there a plan for translation or recruiting study staff who speak the language to double code these studies?
- Additional intervention variables might be interesting (e.g., stimulus type [words, faces], whether the stimuli were personalized for the participant, number of trials, number of sessions)
- Will the authors calculate effect sizes for CBM on social anxiety at follow-up?
- Updating the review every 3 months may be challenging (i.e., conducting a search, exporting the articles identified, abstract review, full text, data abstraction, and risk of bias is a lot to accomplish in 3 months). Perhaps bi-annually would be more effective, while still keeping the review current?
- Our team has found that our risk of bias rating varies based on the outcome measure. For studies with multiple continuous outcomes (e.g., clinician rated and self-report) will multiple risk of bias ratings be calculated? Will clinician-administered outcomes be prioritized?

RESULTS

- Please define REML (Pairwise meta-analysis section).
- Whether in the results or intro/method, I think it would be useful to describe what Network Meta-Analysis accomplishes (i.e., allows us to compare interventions for which there may not be head-to-head trials, if they have been tested against similar comparators)
- I am surprised that the authors are choosing to conduct pairwise meta-analysis across all CBM interventions as there is considerable heterogeneity in these interventions and the proposed intervention target for each.
- Will publication bias be evaluated?
- How will the authors group/categorize the difference types of CBMs for use in the network meta-analysis?
- Applying machine learning to study selection is a big undertaking – one that the authors mention very briefly but do not describe adequately to understand the methods they mean to use.

Overall, I believe the authors have a unique opportunity to provide a valuable resource to the field of CBM, yet there is no mention of making the systematic review available to other study teams (perhaps after an embargo period) or disseminating the proposed machine learning tools. Applying FAIR data principles to the proposed study could greatly increase its impact.

Is the study design appropriate for the research question?

Yes

Is the rationale for, and objectives of, the study clearly described?

Partly

Are sufficient details of the methods provided to allow replication by others?

Yes

Are the datasets clearly presented in a useable and accessible format?

No

Reviewer Expertise:

My research focuses on cognitive dysfunction in PTSD and related conditions with an emphasis on attentional bias and cognitive bias modification. I also work on several large study-level data repositories (i.e., PTSD Repository, Suicide Prevention Trials Database), which are similar to the living review in the proposed study.

I confirm that I have read this submission and believe that I have an appropriate level of expertise to confirm that it is of an acceptable scientific standard, however I have significant reservations, as outlined above.

# Reviewer report: Reviewer response for version 1

**DOI:** 10.21956/wellcomeopenres.25657.r110655

**Published:** 2024-12-09

**Authors:** David M. Clark

**Affiliations:** 1 University of Oxford, Oxford, UK

Cognitive bias modification is a field that is somewhat controversial due to several failures to replicate beneficial effects of various cognitive bias interventions on social anxiety.

 A comprehensive meta-analysis could potentially provide clarity. The proposed meta-analysis has many strengths including: 1) looking separately at studies with people who have a diagnosis of social anxiety disorder (SAD) and those who score high on relevant scales without necessarily being diagnosed and 2) breaking down the diverse category of cognitive bias modification into potentially more informative subcategories.

 It is suggested the study will be a "living review" with new literature searches conducted every three months and analyses being repeated when new studies become available, I could not find any justification for the every three months feature. Is this really a field where so many studies are being conducted that a re-analysis has to be done every three months? Wouldn't a repeat after 12 months be adequate? Also is it feasible to run complex procedures like a network meta-analysis every 3 months?

 There are some other aspects of the design that can be questioned.

 1) Cognitive bias interventions will be divided into 7 sub-categories. How are these sub-categories defined and how will the investigators check that they have allocated a study to the correct sub-category?  Will they share their category definitions with the authors of included studies and check with the authors that the type of intervention in their study has been correctly identified? This a major current issue with  meta-analytic teams are often being criticised for their classification of interventions and a lack of transparency about the process.

 2) Selection of outcome measures. There is likely to be considerable variation in the measures of social anxiety used in different studies. I am not convinced that the authors' way of dealing with this is optimal. Instead of the priority list that they propose, one might take an average of  standardised scores on all appropriately validated measures of social anxiety. 

 3) Several important aspects of trial design aren't mentioned and so may not be taken into account. Nowadays it is generally agreed that the design of a trial (including the proposed primary outcome measures and analytic procedures ) should be pre-registered. I could not see evidence that this crucial variable will be taken into account. 

 4) Comparison with other active psychological therapies. There are a number of well-validated psychological therapies for SAD that are in widespread use. If the review is to change clinical practice it will need to also look at how various forms of CBM compare with existing properly specified alternatives. This doesn't seem to be included in the scope (lumping all alternatives together as TAU is not acceptable)

Is the study design appropriate for the research question?

Partly

Is the rationale for, and objectives of, the study clearly described?

Yes

Are sufficient details of the methods provided to allow replication by others?

Partly

Are the datasets clearly presented in a useable and accessible format?

Yes

Reviewer Expertise:

Treatment of social anxiety disorder

I confirm that I have read this submission and believe that I have an appropriate level of expertise to confirm that it is of an acceptable scientific standard, however I have significant reservations, as outlined above.
